# Comparing effects of two higher intensity feedback interventions with simple feedback on improving staff communication in nursing homes—the INFORM cluster-randomized controlled trial

**DOI:** 10.1186/s13012-020-01038-3

**Published:** 2020-09-10

**Authors:** Matthias Hoben, Liane R. Ginsburg, Adam Easterbrook, Peter G. Norton, Ruth A. Anderson, Elizabeth A. Andersen, Anne-Marie Boström, Lisa A. Cranley, Holly J. Lanham, Lori E. Weeks, Greta G. Cummings, Jayna M. Holroyd-Leduc, Janet E. Squires, Adrian S. Wagg, Carole A. Estabrooks

**Affiliations:** 1 Faculty of Nursing, University of Alberta, 11405 87 Avenue, Edmonton, Alberta T6G 1C9 Canada; 2 School of Health Policy & Management, Faculty of Health, York University, Toronto, Ontario M3J 1P3 Canada; 3 Cumming School of Medicine, University of Calgary, Calgary, Alberta T2N 4 N1 Canada; 4 School of Nursing, University of North Carolina, Chapel Hill, NC 27599-7460 USA; 5 School of Nursing, Thompson Rivers University, Kamloops, British Columbia V2C 0C8 Canada; 6 Division of Nursing, Department of Neurobiology, Care Sciences and Society, Karolinska Institutet, Huddinge, SE-104 35 Stockholm, Sweden; 7 Lawrence S Bloomberg Faculty of Nursing, University of Toronto, Toronto, Ontario M5T 1P8 Canada; 8 University of Texas Health Science Center San Antonio, University of Texas, San Antonio, TX 78229 USA; 9 School of Nursing, Faculty of Health, Dalhousie University, Halifax, Nova Scotia B3H 4R2 Canada; 10 School of Nursing, Faculty of Health Sciences, University of Ottawa, Ottawa, Ontario K1H 8 M5 Canada; 11 Division of Geriatric Medicine, Department of Medicine, Faculty of Medicine & Dentistry, University of Alberta, Edmonton, Alberta T6G 2P4 Canada

**Keywords:** Nursing homes, Care aides, Formal communication, Audit and feedback, Randomized controlled trial, Quality improvement

## Abstract

**Background:**

Effective communication among interdisciplinary healthcare teams is essential for quality healthcare, especially in nursing homes (NHs). Care aides provide most direct care in NHs, yet are rarely included in formal communications about resident care (e.g., change of shift reports, family conferences). Audit and feedback is a potentially effective improvement intervention. This study compares the effect of simple and two higher intensity levels of feedback based on goal-setting theory on improving formal staff communication in NHs.

**Methods:**

This pragmatic three-arm parallel cluster-randomized controlled trial included NHs participating in TREC (translating research in elder care) across the Canadian provinces of Alberta and British Columbia. Facilities with at least one care unit with 10 or more care aide responses on the TREC baseline survey were eligible. At baseline, 4641 care aides and 1693 nurses cared for 8766 residents in 67 eligible NHs. NHs were randomly allocated to a simple (control) group (22 homes, 60 care units) or one of two higher intensity feedback intervention groups (based on goal-setting theory): basic assisted feedback (22 homes, 69 care units) and enhanced assisted feedback 2 (23 homes, 72 care units). Our primary outcome was the amount of formal communication about resident care that involved care aides, measured by the Alberta Context Tool and presented as adjusted mean differences [95% confidence interval] between study arms at 12-month follow-up.

**Results:**

Baseline and follow-up data were available for 20 homes (57 care units, 751 care aides, 2428 residents) in the control group, 19 homes (61 care units, 836 care aides, 2387 residents) in the basic group, and 14 homes (45 care units, 615 care aides, 1584 residents) in the enhanced group. Compared to simple feedback, care aide involvement in formal communications at follow-up was 0.17 points higher in both the basic ([0.03; 0.32], *p* = 0.021) and enhanced groups ([0.01; 0.33], *p* = 0.035). We found no difference in this outcome between the two higher intensity groups.

**Conclusions:**

Theoretically informed feedback was superior to simple feedback in improving care aides’ involvement in formal communications about resident care. This underlines that prior estimates for efficacy of audit and feedback may be constrained by the type of feedback intervention tested.

**Trial registration:**

ClinicalTrials.gov (NCT02695836), registered on March 1, 2016

Contributions to the literature

- This is the first study to rigorously test and compare multiple feedback interventions based on robust goal-setting theory in nursing homes and addresses calls for head-to-head audit and feedback assessments.
- Higher intensity feedback approaches with goal setting and written action plans are more effective in improving formal team communications than standard approaches.
- Both the higher and the lower intensity feedback strategies in the intervention arms were equally effective.
- Theory-based feedback, as used in this study, can improve formal communications among care teams—an outcome that is difficult to change, yet crucial to quality and safety of resident care.

## Background

Effective communication in interdisciplinary healthcare teams is key to quality and safety in healthcare. In 2001, the US Institute of Medicine identified a lack of interdisciplinary communication and collaboration as a major reason for system-wide quality and safety issues [1]. Communication issues are the root cause of one in five sentinel events in healthcare [2], were responsible for a third of all malpractice claims in the USA in 2009–2013, and resulted in more than 1700 deaths and $1.7 billion in avoidable costs [3]. International studies demonstrate that improved communication in interdisciplinary healthcare teams improves patients’ depressive symptoms [4], reduces risks of postoperative complications and mortality [5], and improves assessment and patient management practices in oncology settings [6]. A recent study in acute care settings showed that enhancing communication between healthcare providers, patients, and their families using formal interactions significantly reduced 7-day readmission rates [7]. In contrast to informal communications (e.g., spontaneous discussions on the hallway), formal communications are planned and scheduled meetings to discuss and make decisions about care. In nursing homes, a systematic review found that better formal team communication improved resident outcomes including responsive behaviors, falls, use of antipsychotics, depressive symptoms, appropriateness of medications, restraint use, nutrition, and pain [8]. However, specific interventions, roles of team members, and implementation processes were often poorly described and evaluated [8]. A significant knowledge gap exists on how to effectively improve team communication, which is a prerequisite for improved patient outcomes [9] and team quality improvement success.

The quality of care in nursing homes is an international source of concern, but increasing regulation, inspection, and research to improve quality have had limited success [10, 11]. Nursing homes are a vital component of the health and social care system, providing 24-h care to people with complex care needs who are unable to live in their own homes. Residents are commonly frail, older, have substantial disability, and up to 70% have a diagnosis of dementia [12–14] (which may be underestimated by more than 10% [15]). With an aging population and policy shifts that support people living in their own homes as long as possible, the needs of the nursing home population have become increasingly complex [16]. In addition, 60–80% of the nursing home workforce are care aides (also called care assistants, personal support workers, or nursing assistants) [17]. This largely unregulated workforce has little formal training and low levels of education and wages, but provides most of the direct care to these complex residents [17]. Care aides have knowledge that no other care provider group has, because they are in protracted, close contact with residents and have an intimate awareness of their care needs and preferences. Their knowledge is key to improving the quality of care and life for frail, vulnerable older nursing home residents [18]. However, research in nursing homes suggests that information exchange between care aides and regulated staff is top-down and that care aides are rarely involved in decisions about resident care [19].

Audit and feedback were identified as an effective intervention to improve care team performance [11, 20, 21]. It involves assessing recipients’ performance and providing them with a summary of their performance over a specified period of time [20, 22]. However, audit and feedback often achieves only marginal gains [20], because studies often (a) are not based on robust theory [23], (b) compare audit and feedback to no intervention, rather than comparing different approaches to audit and feedback [22], and (c) focus on education alone [24], rather than including concrete tools and strategies for achieving improvement [25]. Our study responds to calls to optimize audit and feedback and maximize its effects by addressing these issues [23]. Furthermore, limited evidence is available on audit and feedback in nursing homes and especially on its effectiveness in improving the inclusion of care aides in formal communications about resident care [26].

TREC (translating research in elder care) is a longitudinal program of applied health services research that since 2007 has collected comprehensive data on nursing home residents, care staff, care units, and facilities [27]. TREC’s mission is to improve the quality of care and quality of life for frail, older nursing home residents, and quality of worklife for their paid caregivers. Feeding back research data to care teams has always been one of TREC’s integral activities [28–31]. To improve formal communication in interdisciplinary care teams and integrate care aides into these formal communications, we further developed our feedback approach and included two higher intensity feedback interventions based on goal-setting theory and compared them to our simple feedback intervention [32], which in this case was our team’s usual feedback approach. Here, we report the effectiveness of a large definitive cluster-randomized controlled trial (INFORM, improving nursing home care through feedback on perfoRMance data) in increasing care aide involvement in formal team communications and decision-making about resident care. Secondary outcomes included care aides’ perception of (a) their work environment (evaluation, social capital, slack time), (b) use of research, (c) quality of worklife (psychological empowerment, job satisfaction), and two indicators of quality of resident care (percent of residents with worsening responsive behaviors and percent of residents with worsening pain). Consistent with MRC guidelines [33] and suggestions by other audit and feedback [23] and quality improvement experts [34], we simultaneously conducted a fidelity study to deepen understanding of the effectiveness results presented here [submitted to Impl Sci as a companion paper to this paper; add reference upon acceptance].

## Methods

### Study design

In a pragmatic three-arm parallel cluster-randomized controlled trial with assessment at baseline and at 12-month follow-up, we compared a simple feedback approach with two higher intensity feedback approaches: basic assisted feedback and enhanced assisted feedback. In contrast to explanatory trials, aiming to assess whether an intervention works under well-defined and highly controlled conditions, pragmatic trials aim to assess whether an intervention is effective under “real-life conditions” that are more complex and less controllable and predictable [35]. Although the primary study outcome focused on the involvement of care aides in formal team communications about resident care, the intervention was directed to care unit managerial teams: care managers, the director of care, and persons who assist them (e.g., clinical educators). We targeted managers because they are the persons who have the power and ability to change the organizational structures and processes required to facilitate care aide involvement in these formal communications. Nursing homes are made up of one or more care units, the organizational subunits where residents receive care by care teams. Quality of care can vary substantially within nursing home care units. Norton et al. [36] demonstrated that improvement strategies targeted at the care unit level are more powerful than those at the facility level. We performed cluster randomization (i.e., all units within a nursing home randomized into the same study arm) to prevent contamination (i.e., spread of the intervention to non-intervention units). Our study intervention was designed to help care teams increase care aide involvement in formal team communications about resident care, to improve quality of communication, worklife for care staff, and resident care.

INFORM was registered (ClinicalTrials.gov Identifier: NCT02695836) and the trial protocol published [26]. We followed CONSORT reporting guidelines for cluster-randomized trials [37].

### Hypotheses

- Basic and enhanced assisted feedback on care aide involvement in formal communications about resident care to managerial teams will increase (a) care aide involvement in formal team communications, (b) quality of resident care, and (c) care aide quality of worklife more strongly than simple feedback.
- Enhanced assisted feedback on care aide involvement in formal communications about resident care to managerial teams will increase (a) care aide involvement in formal team communications, (b) quality of resident care, and (c) care aide quality of worklife more strongly than basic assisted feedback.

### Setting

Nursing homes participating in TREC (translating research in elder care, a longitudinal program of applied health and care research), took part in this study [27]. At INFORM inception, TREC included 75 urban nursing homes with 9613 residents across the Western Canadian provinces of Alberta and British Columbia, randomly selected from the overall urban nursing home population and stratified by health region, ownership type, and size. TREC facilities participate in a longitudinal observational study that generates a comprehensive dataset of residents, care staff, care unit, and facility outcomes. We used data from our September 2014–May 2015 wave of primary data collection to assess baseline outcomes, then carried out our INFORM intervention in the subset of TREC facilities participating in INFORM, and then carried out our January 2017–December 2017 wave of primary data collection to assess follow-up outcomes.

### Participants

Table 1 lists the inclusion and exclusion criteria for facilities and care units. Only nursing homes that participated in the TREC observational study were included because our trial outcomes were available for these facilities. Only facilities with at least one care unit with 10 or more care aide responses on the TREC baseline survey were eligible, for stable, valid, and reliable aggregation of study outcomes at the unit level [38]. We excluded facilities if we were unable to assign surveys to their care units, which made unit-level analyses impossible in those sites. We only included care units with an identifiable care manager or leader. At baseline, 4641 care aides and 1693 nurses cared for a total of 8766 residents in 67 eligible nursing homes.

**Table 1:** Inclusion and exclusion criteria for facilities and care units

	Inclusion criteria	Exclusion criteria
**Facilities**	• Participating in the TREC observational study • Located in one of four health regions in Alberta (Edmonton or Calgary) or British Columbia (Fraser Health or Interior Health) • Had at least one care unit with 10 or more care aide responses to our survey that we used to assess organizational context and staff outcomes at baseline	• Surveys collected in this facility could not be assigned to a care unit (as defined by TREC within this facility)
**Care units**	• Located within a facility participating in the TREC observational study in Alberta or British Columbia • Had 10 or more care aide responses to the TREC survey that we used to assess organizational context and staff outcomes at baseline • Identified a care manager who led this unit	• Care unit located in a nursing home participating in TREC, but care unit was not eligible for the TREC study (unit providing sub-acute care, rehabilitation, assisted living, day care, etc.)

### Randomization and masking

To avoid contamination, we randomized at the facility level. All care unit managerial teams within a facility received the same feedback intervention. Using stratified permuted block randomization, an independent person not involved in this study assigned eligible nursing homes to one of three study arms. Study arm allocation was determined by assigning computer-generated random numbers to facilities. Randomization was stratified by health region (Edmonton or Calgary Health Zones in Alberta, Fraser, or Interior Health Regions in British Columbia) to account for regional policy differences that might influence structures, processes, or outcomes in participating facilities.

A regional project coordinator in each health region obtained additional written informed consent from facilities randomized to the basic and enhanced assisted feedback arms. These facilities were offered additional feedback—coordinators explained to managers that they would receive specific extra feedback as part of the intervention—but we blinded managers to the fact that there were additional study arms. Facilities in different study arms received different recruitment materials (information sheets and informed consents) and attended different types of workshops. Coordinators organized intervention workshops, invited managerial teams to workshops, and disseminated workshop materials to teams. Coordinators could not be blinded to study arm allocation in their region, but we blinded each coordinator to how facilities were allocated in all other regions. Coordinators were requested not to reveal to anyone how their facilities were allocated. To inform a coordinator about study arm allocation of facilities in their region, we used a secure online platform (https://www.igloosoftware.com/) that could only be accessed by the coordinator, the person who carried out randomization, and the system administrator.

People who delivered the intervention and monitored intervention fidelity were (a) a facilitator and a study investigator who attended every workshop and (b) additional investigators, trainees, decision-makers, and study staff (varied by region and time of intervention). Workshop contents, activities, duration, materials, and format differed between basic and enhanced assisted feedback groups (described below). The simple feedback group (our control or usual care arm) received tailored feedback reports only 2-3 months after the baseline data collection. We consider simple feedback “usual care” because all TREC homes receive this kind of feedback after each wave of data collection [28–31], regardless of whether they participate in an intervention study or any other TREC activity. To deliver the correct intervention to each study group and to monitor correct fidelity criteria, persons involved in these activities could not be blinded to study arm allocation. However, they were requested not to talk about facilities by name or share information that could identify a facility or its allocation status. None of these persons took part in data analyses. Before workshops, each facility agreed to not share workshop tools with other managers or facilities during the study.

Data analyses were carried out by analysts not involved in intervention delivery or data collection. These analysts worked with de-identified data sources (surveys, interview, and focus group transcripts) and data sets. A research assistant not involved in INFORM assigned a unique random ID to each participant, care unit, and facility in all data sources before processing, cleaning, and analysis. Random IDs were generated by the same person who randomized facilities. Only that person, the research assistant, and the TREC managing director had access to the de-identification list, and they were all required to keep this information confidential.

### Interventions

**Fig. 1:**
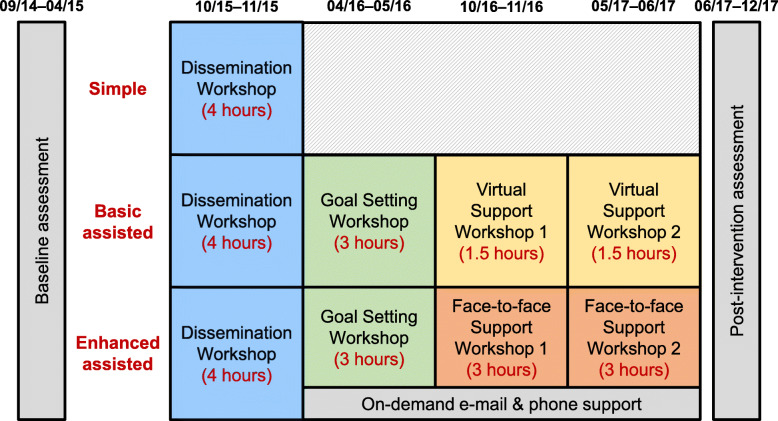
Study arms

The study had three arms (Fig. 1). The audit component of this study is reflected by the baseline data collections for care units in all three study arms. We used baseline data on a care unit’s involvement of care aides in formal communications about resident care and fed these data back to care units. The control or usual care group received simple feedback only: a feedback report to each facility with information on resident and care staff outcomes. This feedback report was discussed in 4-h face-to-face dissemination workshops. In addition to simple feedback, the two higher intensity groups received a more focused feedback report on care aides’ involvement in formal communications about resident care. Results were discussed in a 3-h in-person goal-setting workshop. Basic assisted feedback participants received two additional 1.5-h web-based support workshops. Enhanced assisted feedback participants received two additional 3-h in-person support workshops and had access to on-demand email and phone support. For the enhanced assisted feedback arm, longer workshops, and in-person contact of participants with peers and the study team aimed to provide a more intense learning experience to improve intervention effectiveness.

#### Goal-setting theory

Our trial protocol contains a detailed description of the theoretical foundations of our intervention [26]. Briefly, audit and feedback interventions based on goal-setting theory are more effective. Participants who set goals and define strategies to achieve them will identify with the goals, perceive them as achievable, and work to achieve them [32]. Both short-term and long-term goals are required. Short-term goals break down the task and enhance self-efficacy and task persistence. Long-term goals keep people accountable. Both performance and learning goals are also required. Learning goals indicate how to make improvements. Performance goals indicate what to achieve [32]. Written action plans hold individuals accountable and are reminders of performance goals [20, 22]. Specifically, managers and their teams attending the basic and enhanced intervention workshops were supported to specify various performance goals related to the improvements targeted on their care units (such as involving care aides in formal team communications about resident care in 80% (vs 10% at baseline) of the formal team meetings on the care unit within the next 3 months). Further, managers specified learning goals related to their own learning (such as improving a manager’s ability to specify measurable performance goals and to routinely measure success in achieving performance goals) and to their care team’s learning (increase the ability and comfort of care aides to speak up in formal team communications about resident care, and increase the team’s acceptance of care aide opinions as valuable contribution).

#### Dissemination workshops

Sites in all three study arms received simple feedback in November 2015. Reports focused on a core set of actionable measures including care aides’ perception of their involvement in formal communications about resident care (formal interactions), data-based feedback on their care unit’s performance (evaluation), connections within their teams (social capital), and slack time. Managerial teams of each facility were then invited to a half-day face-to-face dissemination workshop where feedback reports were discussed. Workshops were led by an experienced professional facilitator, hired by the study team. After a senior researcher of the research team presented reports, managerial teams participated in small group discussions to interpret results, identify improvement areas, and think about improvement strategies. Workshops did not set specific goals but gave simple instructions on interpreting reports and planning improvement strategies.

#### Goal-setting workshops

In June 2016, care unit managers and their teams in the basic and enhanced assisted feedback groups participated in a face-to-face goal-setting workshop. We held separate workshops for basic and enhanced assisted feedback groups in each of the four health regions. Each unit received a package of goal-setting workshop materials 1 week before the workshop: a feedback report on the care unit’s data (formal interactions, evaluation, social capital, slack time) and a goal-setting workbook summarizing details of the INFORM study, defining key concepts, and outlining the goal-setting approach. Managers were encouraged to bring care staff (care aides, nurses, allied health providers) to the workshops. Workshops used small group activities such as reflecting on data, establishing a series of specific and measurable learning and performance goals, and identifying measures and tools to track goal achievement. Participants generated an action plan and received instructions on how to track goal progress and how to report back at the support workshops.

#### Support workshops

In November 2016, basic assisted feedback participants attended a 1.5-h virtual support workshop via a web-based conference platform. Enhanced assisted feedback participants attended a 3-h face-to-face support workshop. Managerial teams reported their progress in implementing goals and described their implementation strategies. They discussed challenges encountered and received support from the study team, regional decision-makers, and their peers in addressing these challenges. A second support workshop with the same content was held in April 2017.

#### On-demand email and phone support

Enhanced assisted feedback teams also had access to on-demand email and phone support from the facilitator throughout the intervention period. The facilitator addressed questions and helped resolve challenges as managerial teams worked toward goal achievement.

#### Process evaluation

We comprehensively evaluated intervention fidelity, implementation, and participant experiences using a mixed-methods approach. Methods and results are reported in a separate publication [submitted to Impl Sci as a companion paper to this paper; add reference upon acceptance].

### Outcomes

Our study outcomes were assessed using data collected in two waves of TREC’s longitudinal observational study [27]—September 2014–May 2015 to assess baseline outcomes before the intervention and January 2017–December 2017 to assess follow-up outcomes after the intervention (Fig. 1). These included care staff use of best practices, quality of worklife (e.g., psychological empowerment, job satisfaction), organizational context, and characteristics of nursing homes and care units (Additional file 1)—all of which were measured using the validated TREC survey [27]. Resident data were obtained from the Resident Assessment Instrument—Minimum Data Set 2.0 (RAI-MDS 2.0) [39]. Nursing homes in health regions participating in TREC are required to assess residents on admission and at least quarterly thereafter and participating TREC homes submit their resident data to TREC on a quarterly basis. Data are used for national reporting.

#### Primary outcome

Our primary outcome was care aides’ self-reported involvement in formal team communications about resident care (formal interactions). *Formal interactions* is one of 10 concepts measured by the Alberta Context Tool, a comprehensively validated tool to assess modifiable features of care unit work environments (details in Additional file 1) [40]. The Alberta Context Tool is embedded within the TREC care aide survey [27], a suite of validated survey instruments completed by computer-assisted structured personal interview. The formal interactions rating consists of four items (rated from 1 = never to 5 = almost always) asking care aides how often, in the last typical month, they participated in the following: team meetings about residents, family conferences, change-of-shift reports, and continuing education (conferences, courses) outside their nursing home. In our psychometric studies [40], we found that the most valid way to generate an overall score is count-based: recoding each item (1 and 2 to 0; 3 to 0.5; 4 and 5 to 1) and summing recoded values (possible range, 0–4).

#### Secondary outcomes

##### Organizational

Using the Alberta Context Tool completed by care aides, we assessed evaluation (feedback of routine data to the unit), social capital, and slack time. Using the TREC unit survey completed by managers, we assessed care unit managerial teams’ responses to major near misses and managers’ organizational citizenship behavior. Using the TREC facility survey completed by Directors of Care, we assessed processes and practices in quality improvement activities. These instruments are described elsewhere [27] and in Additional file 1.

##### Staff

Using the TREC care aide survey, we assessed care aides’ use of best practice, psychological empowerment, job satisfaction, and individual staff attributes (Additional file 1).

##### Residents

Using the RAI-MDS 2.0 data, we assessed two practice-sensitive (modifiable by care staff) quality indicators [41]: residents with worsening pain and residents with declining behavioral symptoms (Additional file 1). These outcomes were chosen because they are two of the most practice sensitive (modifiable by care staff) [41] quality indicators and both, responsive behaviors and pain are considered among the most burdensome resident outcomes [42].

### Statistical analyses

#### Sample size calculation

Full details of the sample size calculation are in our trial protocol [26]. Assumed effect sizes of the formal interactions score were *β*_1_ = 0.2 in the simple feedback group, *β*_2_ = 0.4 in the basic assisted feedback group, and *β*_3_ = 0.6 in the enhanced assisted feedback group. An adapted simulation-based approach [43] suggested that 12 facilities per study arm (with on average three units per facility) were required to detect the assumed effects with a statistical power of 0.90. To allow for attrition and effects smaller than the assumed ones, we invited all eligible units in the 67 eligible facilities in Alberta and British Columbia to participate in this study.

#### Statistical approach

We used SAS® 9.4 for all statistical analyses. Using descriptive statistics, between study arms we compared baseline characteristics of nursing homes, care units, participants in our first intervention workshop, and care aides working on participating units. To assess the effects of interventions on our primary outcome (formal interactions) and secondary staff outcomes, we ran mixed effects regression models with random intercepts for care unit and facility levels, and a random effect for care aides responding to our survey at both baseline and follow-up. We adjusted the model for the three stratification variables of the TREC facility sample (region, owner-operator model, and facility size); baseline differences of the dependent variables; care aides’ sex, age, and first language (English yes/no); and care unit staffing (total care hours per resident day and percentage of total hours per resident day provided by care aides). Intra-cluster correlation (ICC) of formal interaction scores within facilities and care units was calculated by dividing the cluster-level variance by the total variance (sum of residual variance, unit-level random intercept, and facility-level random intercept). For secondary resident outcomes (percent of residents on a care unit whose behavior worsened and percent of residents on a care unit whose pain worsened), we ran mixed effects regression models with a facility-level random intercept. These models were adjusted for facility characteristics (region, owner-operator model, and facility size); baseline differences of the dependent variables; and care unit staffing. We carried out an intention-to-treat analysis. A care unit was considered to be adherent with the intervention if at least one representative of this unit attended the goal-setting workshop and at least one of the two support workshops.

### Public and patient involvement

TREC is a program of integrated knowledge translation research [44]. Throughout all projects, TREC partners with researchers, trainees, policymakers, owner-operators, care staff, people in need of care, and their family/friend caregivers in all phases of the research process. In INFORM, these stakeholders were co-applicants on the research grant that funded the study, were team members of working groups and committees that carried out the study, and were involved in discussions on study results and their interpretation.

## Results

**Fig. 2:**
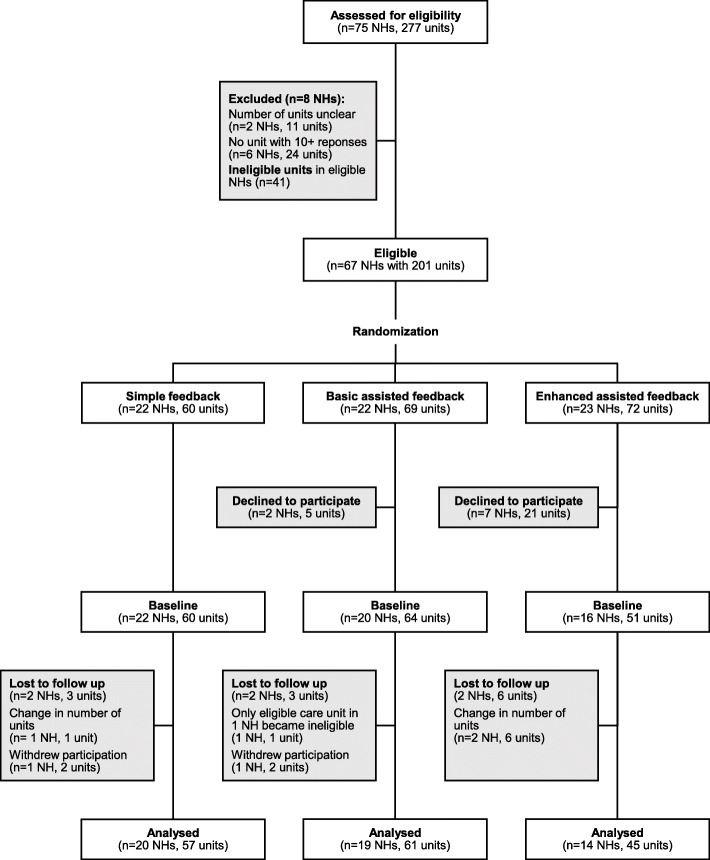
Trial profile

Between November 1 and 15, 2015, we assessed 75 nursing homes with 277 care units for eligibility. We randomized each of the 67 eligible nursing homes (201 eligible care units) into one of the three study arms (Fig. 2). Two nursing homes with 5 eligible care units declined participation in the basic assisted feedback arm and seven nursing homes with 21 eligible care units declined participation in the enhanced assisted feedback arm. Two nursing homes (three eligible units) in each of the simple and basic assisted feedback arms and two nursing homes (six eligible care units) in the enhanced assisted feedback arm were not included in final analyses because no follow-up data were available. One nursing home (six eligible care units) in the basic assisted feedback arm did not participate in any intervention workshops, but we included this facility in intention-to-treat analyses.

Table 2 presents baseline characteristics of included facilities, care units, and participants attending the first intervention workshop (goal-setting workshop), and of care aides by study arm. Facilities, care units, and care aides had similar characteristics at baseline in all study arms. Numbers of participants attending the first intervention workshop (predominantly managers) were also comparable. Care aides were predominantly female and older than 40 years. They had worked for 10–11 years on average as a care aide and had 5–6 years of experience on their current unit.

**Table 2:** Facility, care unit, goal-setting workshop participant, and care aide characteristics at baseline by study arm

	Simple feedback (control)	Basic assisted feedback	Enhanced assisted feedback
**Facility sample**			
**Number of facilities**	20	19	14
**Region**			
Calgary	5 (25.0%)	4 (21.1%)	3 (21.4%)
Edmonton	4 (20.0%)	5 (26.3%)	3 (21.4%)
Fraser	7 (35.0%)	6 (31.6%)	6 (42.9%)
Interior	4 (20.0%)	4 (21.1%)	2 (14.3%)
**Size**			
Small	6 (30.0%)	6 (31.6%)	3 (21.4%)
Medium	5 (25.0%)	7 (36.8%)	5 (35.7%)
Large	9 (45.0%)	6 (31.6%)	6 (42.9%)
**Ownership**			
Public	5 (25.0%)	5 (26.3%)	3 (21.4%)
Voluntary	9 (45.0%)	8 (42.1%)	8 (57.1%)
Private	6 (30.0%)	6 (31.6%)	3 (21.4%)
**Number of care units,** M ± SD	2.9 ± 1.7	3.2 ± 2.3	3.2 ± 2.0
**Care unit sample**			
**Number of care units**	57	61	45
**Unit type**			
General long-term care		2 (3.3%)	
Regular dementia	10 (17.5%)	11 (18.0%)	10 (22.2%)
Secure dementia	42 (73.7%)	38 (62.3%)	28 (62.2%)
Regular mental health/psychiatric	4 (7.0%)	7 (11.5%)	7 (15.6%)
Other	1 (1.8%)	3 (4.9%)	
**Unit staffing**, M ± SD hours per resident day			
Care aides	2.0 ± 0.7	2.3 ± 0.7	2.3 ± 0.6
Licensed practical nurses	0.8 ± 0.6	0.5 ± 0.4	0.5 ± 0.2
Registered nurses	0.6 ± 0.6	0.4 ± 0.2	0.3 ± 0.3
Total staffing	3.3 ± 1.6	3.2 ± 1.0	3.1 ± 0.8
**Goal-setting workshop participant sample**			
**Number of participants**		60	37
**Region**			
Calgary		15 (25.0%)	4 (4.1%)
Edmonton		25 (41.7%)	7 (7.2%)
Fraser		12 (20.0%)	21 (21.7%)
Interior		8 (13.3%)	5 (5.2%)
**Participant role**			
Manager		37 (62.7%)	22 (59.5%)
Regulated care staff		13 (22.0%)	8 (21.6%)
Care aide		1 (1.7%)	3 (8.1%)
Other		9 (13.6%)	4 (10.8%)
**Years worked in facility,** M ± SD		7.8 ± 7.4	8.0 ± 8.1
**Care aide sample**			
**Number of care aides**	751	836	615
**Females**	683 (90.9%)	764 (91.4%)	553 (89.9%)
**Age category**			
< 25 years	20 (2.7%)	13 (1.6%)	35 (5.7%)
25–34 years	146 (19.4%)	118 (14.1%)	107 (17.4%)
35–44 years	216 (28.8%)	259 (31.0%)	178 (28.9%)
45–54 years	203 (27.0%)	262 (31.2%)	182 (29.6%)
> 54 years	166 (22.1%)	184 (22.0%)	113 (18.4%)
**English as second language**	453 (60.3%)	580 (69.5%)	363 (59.0%)
**Number of care homes working in**			
1	551 (73.7%)	554 (66.4%)	474 (77.1%)
2	172 (23.0%)	256 (30.7%)	124 (20.2%)
3	19 (2.5%)	22 (2.6%)	13 (2.1%)
4	5 (0.7%)	2 (0.2%)	4 (0.7%)
5	1 (0.1%)		
**Shift worked most often**			
Day	351 (46.7%)	449 (53.8%)	286 (46.6%)
Evening	314 (41.8%)	298 (35.7%)	262 (42.7%)
Night	86 (11.5%)	88 (10.5%)	66 (10.7%)
**High school degree**	712 (94.8%)	780 (93.5%)	574 (93.3%)
**Care aide certificate**	696 (92.7%)	761 (91.1%)	566 (92.0%)
**Years worked on unit**, M ± SD	5.4 ± 5.7	6.6 ± 6.5	5.0 ± 4.9
**Years worked as care aide**, M ± SD	10.6 ± 8.9	11.5 ± 8.8	10.3 ± 8.7
**Hours worked in last 2 weeks**, M ± SD	71.1 ± 20.5	71.6 ± 22.4	68.3 ± 19.8

Table 3 presents the number of intervention workshops that each facility and care unit attended.

**Table 3:** Facility-level and care unit-level workshop attendance

	Basic assisted feedback	Enhanced assisted feedback
**Facility attendance**		
Number of facilities	19	14
Non-adherence		
Attended no workshops	0 (0.0%)	1 (7.1%)
GSW* only	1 (5.3%)	0 (0.0%)
Partial adherence		
GSW + SW1*	4 (21.1%)	2 (14.3%)
GSW + SW2	0 (0.0%)	1 (7.1%)
Full adherence		
All three workshops	14 (73.7%)	10 (71.4%)
**Care unit attendance**		
Number of care units	61	45
Non-adherence		
Attended no workshops	9 (14.8%)	6 (13.3%)
GSW only	4 (6.6%)	0 (0.0%)
Partial adherence		
GSW + SW1	12 (19.7%)	3 (6.7%)
GSW + SW2	0 (0.0%)	5 (11.1%)
Full adherence		
All three workshops	36 (59.0%)	31 (68.9%)

*GSW goal-setting workshop, *SW* support workshop

Table 4 presents the study outcome scores at baseline and follow-up.

**Table 4:** Study outcomes (M ± SD) by study arm at baseline and follow-up

	Baseline			Follow-up		
	Simple feedback (control)	Basic assisted feedback	Enhanced assisted feedback	Simple feedback (control)	Basic assisted feedback	Enhanced assisted feedback
**Number of care aides**	751	836	615	767	808	578
**Number of care units**	57	61	45	57	61	45
**Primary outcome**						
Formal Interactions	1.35 ± 0.79	1.48 ± 0.80	1.42 ± 0.84	1.40 ± 0.81	1.56 ± 0.80	1.53 ± 0.78
**Secondary staff outcomes**						
Evaluation	3.66 ± 0.63	3.77 ± 0.59	3.66 ± 0.61	3.74 ± 0.61	3.82 ± 0.54	3.76 ± 0.62
Social capital	4.05 ± 0.50	4.10 ± 0.48	4.07 ± 0.48	4.02 ± 0.49	4.09 ± 0.51	4.07 ± 0.48
Slack time	3.49 ± 0.86	3.57 ± 0.81	3.43 ± 0.83	3.49 ± 0.91	3.53 ± 0.82	3.47 ± 0.82
Conceptual research use	4.09 ± 0.73	4.19 ± 0.70	4.03 ± 0.74	4.05 ± 0.77	4.13 ± 0.70	4.03 ± 0.77
Instrumental research use	4.63 ± 0.58	4.62 ± 0.59	4.62 ± 0.60	4.62 ± 0.58	4.59 ± 0.58	4.62 ± 0.56
Psychological empowerment, meaning	4.57 ± 0.48	4.58 ± 0.48	4.60 ± 0.49	4.57 ± 0.48	4.53 ± 0.50	4.54 ± 0.48
Job satisfaction	4.24 ± 0.62	4.33 ± 0.57	4.22 ± 0.62	4.25 ± 0.65	4.29 ± 0.63	4.18 ± 0.64
**Secondary resident outcomes**						
Percent of residents whose responsive behaviors worsened	10.9 ± 6.6	12.2 ± 6.3	13.0 ± 5.6	10.5 ± 5.8	11.1 ± 6.1	13.1 ± 6.6
Percent of residents whose pain worsened	10.6 ± 5.8	10.2 ± 6.9	8.9 ± 4.0	10.6 ± 5.4	10.8 ± 6.1	7.6 ± 5.8

Notes: Possible ranges of scores are 0-4 for formal interactions, 1-5 for all secondary staff outcomes, and 0-100 for secondary resident outcomes. Higher scores reflect better outcomes in primary and secondary staff outcomes

### Primary outcome

There was a statistically significant increase in care aides’ attendance at formal team communications about resident care in both the basic and enhanced assisted feedback arms compared with the control arm, as measured by the multiply adjusted model (Table 5). However, this outcome was not different between the basic and enhanced assisted feedback arms. Unit-level ICCs (95% CIs) of this outcome were 0.0437 (0.0263, 0.0860) at baseline and 0 at follow-up. Facility-level ICCs (95% CIs) were 0.0510 (0.0276, 0.1287) at baseline and 0.0453 (0.0274, 0.0920) at follow-up.

**Table 5:** Adjusted mean differences of study outcomes at follow-up based on mixed effects models

	Adjusted difference in least-square means* [95% CI] ***p*** value		
	Basic assisted feedback–simple feedback	Enhanced assisted feedback–simple feedback	Enhanced assisted feedback–basic assisted feedback
**Number of care aides**			
**Number of care units**			
**Primary outcome**			
Formal interactions	**0.17 [0.03; 0.32] 0.021**	**0.17 [0.01; 0.33] 0.035**	0.004 [−0.16; 0.16] 0.961
**Secondary staff outcomes**			
Evaluation	0.09 [−0.01; 0.20] 0.083	0.03 [−0.08; 0.14] 0.587	−0.06 [−0.05; 0.18] 0.292
Social capital	**0.09 [0.01; 0.17] 0.026**	0.07 [−0.01; 0.15] 0.093	−0.02 [−0.10; 0.07] 0.686
Slack time	0.15 [−0.02; 0.31] 0.078	0.03 [−0.15; 0.20] 0.762	−0.09 [−0.30; 0.06] 0.190
Conceptual research use	0.06 [−0.05; 0.17] 0.270	−0.02 [−0.14; 0.10] 0.724	−0.08 [−0.20; 0.04] 0.171
Instrumental research use	−0.009 [−0.08; 0.06] 0.808	0.003 [−0.08; 0.08] 0.941	0.01 [−0.07; 0.09] 0.766
Psychological empowerment, meaning	−0.03 [−0.09; 0.03] 0.373	−0.03 [−0.09; 0.04] 0.437	0.002 [−0.07; 0.07] 0.953
Job satisfaction	0.07 [−0.03; 0.16] 0.179	−0.05 [−0.16; 0.05] 0.301	−**0.12 [**−**0.22;** −**0.02] 0.025**
**Secondary resident outcomes**			
Percent of residents whose responsive behaviors worsened	0.02 [−2.4; 2.5] 0.989	**2.9 [0.3; 5.6] 0.031**	**2.9 [0.2; 5.7] 0.036**
Percent of residents whose pain worsened	0.1 [−2.6; 2.8] 0.935	−1.6 [−4.6; 1.3] 0.278	−1.8 [−4.8; 1.3] 0.256

Bold, *p* < 0.05

### Secondary outcomes

Care aides’ social capital scores were higher in the basic assisted feedback arm than in the simple feedback (control) arm. Care aide job satisfaction in the enhanced assisted feedback arm was lower than in the basic assisted feedback arm. There were no differences between arms in other secondary staff outcomes. Enhanced assisted feedback care units had higher rates of residents whose responsive behavior worsened over the study than simple and basic assisted feedback care units. We found no differences between study arms at follow-up in the proportion of residents whose pain worsened.

Characteristics of included and excluded facilities were not different, and neither were characteristics of care units nor care aides in included versus excluded facilities. A per-protocol analysis (only including care units who sent a representative to all workshops [analysis 1] and care units who attended at least 2 workshops [analysis 2]) did not alter the results and conclusions of our intention-to-treat analysis.

## Discussion

With the goal of improving communication among team members in nursing homes and working with care unit managerial teams in nursing homes, we compared simple (usual care) feedback with one workshop and no goal setting or action plans to two higher intensity feedback processes that included goal setting, generation of action plans, and two follow-up workshops. We found that the higher intensity feedback processes were more effective in increasing care aide involvement in formal communications about resident care (our primary outcome). However, we found no difference in our primary outcome between the two higher intensity intervention groups, even though one group received 3-h in-person support workshops and the other group received 1.5-h web-based support workshops.

Our study first and foremost demonstrates an effective and practical strategy for improving communication among team members in nursing homes, specifically, for enabling and supporting reciprocal communication between unregulated and regulated care staff. Such communication strategies are essential to improving quality and safety in nursing homes and our study offers an achievable pathway to do so. Improved communication is not only an essential quality improvement strategy, it offers benefits to the workforce, specifically improved quality of worklife, here assessed by the construct of social capital. The unregulated workforce in nursing homes is woefully understudied [45–47] and a rising inability of supply to meet demand, as well as, high levels of concern about the conditions of work are urgent international issues [48–50]. Information exchange between care aides and regulated staff is top-down and care aides are rarely involved in decisions about resident care [19, 51, 52]. As essential but highly stressed components of the health and social care systems, nursing homes should be a major focus of implementation and improvement strategies designed to enhance not only quality of care but importantly quality of life for their highly vulnerable population of older adults, the majority with dementia.

In this study, we designed and tested feedback interventions from robust theory on goal setting and compared different interventions head to head. In line with systematic reviews [11, 20, 21], we found that audit and feedback are effective in changing the behaviors of care staff. The most recent Cochrane review on audit and feedback [20] found a weighted median absolute improvement in desired practices of 1.3% (interquartile range; IQR 1.3–28.9%). A systematic review on audit and feedback in dementia care settings reported a non-weighted median absolute improvement of 17% (IQR 0.5–50%) [21]. Our absolute improvement in the formal interactions score for our basic and enhanced assisted feedback groups was 6.4%, comparable to the two reviews and in keeping with the idea that incremental effects of audit and feedback modifications are likely to be small, but important [23].

Our findings contribute knowledge about how to optimize audit and feedback by demonstrating that combining audit and feedback with goal setting and an action plan is more effective than simply feeding back data. However, longer workshops (3-h in the enhanced vs 1.5-h in the basic assisted feedback group) and in-person versus web-based delivery did not further improve the effectiveness of our intervention. This means that significant improvements are possible with a less intense (and therefore less costly) intervention. The web-based workshops in the basic feedback intervention may have been more attractive to care teams (shorter and do not require travel), offsetting the additional learning effect of the more intense enhanced feedback intervention. This may also explain why more facilities declined during the recruitment phase to participate in the enhanced than in the basic feedback group—a concern that 3-h in-person workshops may be too demanding. Finally, how well a facility enacted the intervention was more important for the success of the intervention than the intensity (i.e., basic versus enhanced arm) of the intervention [submitted to Impl Sci as companion paper to this paper; add reference upon acceptance].

Our basic feedback intervention (compared to simple feedback) improved connections among care team members (social capital). However, job satisfaction was lower with enhanced than with basic feedback and our enhanced feedback intervention (compared to simple and basic feedback) was associated with a higher rate of residents with dementia whose behavior worsened. It is possible that our most intense intervention raised care team members’ awareness of behavioral changes, which may have increased their reporting of these outcomes. However, the baseline rates of worsening behavior were already higher in the enhanced group than in the other study groups, and in each group, rates remained relatively stable pre and post-intervention (Table 4). Other secondary study outcomes did not differ between study arms at follow-up. It is possible that, as Wensing and Grol [9] recently highlighted, for organizational and system change to affect resident outcomes, more time is required than is typically available in a research project, and there are many steps (and thus increased time required) from implementation to patient outcome. Studies typically do not assess intermediate steps from implementation to outcome nor are they typically funded for sufficiently long periods to capture changes in patient outcomes. Nonetheless, our study responds to their call to carefully select implementation strategies that fit the problems to be solved, base the intervention on robust theory and evidence, systematically involve stakeholders, and use rigorous study designs and outcome measures that will respond to the intervention.

### Strengths and weaknesses of the study

A strength of our study is that it was systematically based on goal-setting theory [32] and robust evidence on the effect of audit and feedback [11, 20–22] on formal interdisciplinary team communication [4–6, 8]. Our study comprehensively reports all required details of the audit and feedback interventions tested [53] and follows recommendations to conduct concurrent process evaluations with complex behavioral trials [33] such as INFORM—a fidelity sub-study [currently under review as a companion paper to this manuscript] can aid with interpretation of the effectiveness results presented here. We used well-validated measures and robust data collection methods in the INFORM trial [27]. Our cluster-randomized design minimized the risk of contamination between study arms. Robust statistical methods accounting for clustering, baseline differences, and repeated measures maximized the validity of our statistical conclusions.

However, cluster-randomization can introduce a risk of selection bias. Seven nursing homes with 26 care units declined participation, but those numbers are small compared with the whole, and characteristics of those non-participating homes did not differ from the characteristics of participating homes. Given the nature of our intervention, blinding was difficult. However, we blinded facility managerial teams and regional study coordinators to the extent possible to minimize this risk of bias. Another risk of bias could arise from including only facilities already participating in TREC. TREC facilities may be more engaged than other facilities, and interactions with TREC study teams during TREC data collections and feedback activities may make these facilities better equipped to improve. Whether we see this effect in nursing homes that have not been exposed to a research program like TREC needs to be examined in future studies. We also need to assess the long-term sustainability of these effects and whether these improvements in communication translate into improved resident quality of care and quality of life. Therefore, we will assess study outcomes again after our next wave of data collection (September 2019–March 2020).

## Conclusions

Our study findings demonstrate that communications in care homes can be improved by providing feedback, guided by goal-setting theory. It also demonstrates that this theory-based intervention is more effective than simple feedback in improving formal care staff communications about resident care. Results highlight the importance of concrete strategies and support mechanisms for front-line managers and teams wishing to change practice. However, they also suggest highly resource-intensive feedback interventions may be unnecessary. Instead, moderate-intensity interventions that take advantage of more economical web-based delivery and communication technologies may be optimal. Consistent with recent suggestions for health systems and researchers to collaborate in “implementation laboratories” to learn how to advance the science of feedback and optimize its use in practice using larger sequential trials [23], we are revising the INFORM intervention for use by health authorities as a routine quality improvement strategy. We plan to evaluate this revised INFORM package and its rollout across all health regions in a Canadian province. Lastly, in terms of generalizability, the feedback and goal-setting model that INFORM is based on has the potential to improve interprofessional communication and other practices designed to improve nursing home care inside and outside Canada.

## Supplementary information

**Additional file 1.** Study measures.

## Data Availability

The data used for this article are housed in the secure and confidential Health Research Data Repository (HRDR) in the Faculty of Nursing at the University of Alberta (https://www.ualberta.ca/nursing/research/supports-and-services/hrdr), in accordance with the health privacy legislation of participating TREC jurisdictions. These health privacy legislations and the ethics approvals covering TREC data do not allow public sharing or removal of completely disaggregated data (resident-level records) from the HRDR, even if de-identified. The data were provided under specific data sharing agreements only for approved use by TREC within the HRDR. Where necessary, access to the HRDR to review the original source data may be granted to those who meet pre-specified criteria for confidential access, available at request from the TREC data unit manager (https://trecresearch.ca/about/people), with the consent of the original data providers and the required privacy and ethical review bodies. Statistical and anonymous aggregate data, the full dataset creation plan, and underlying analytic code associated with this paper are available from the authors upon request, understanding that the programs may rely on coding templates or macros that are unique to TREC.

## Abbreviations

INFORM: Improving Nursing Home Care Through Feedback On PerfoRMance Data
TREC: Translating Research in Elder Care
