# Supplementary material for: Comparing effects of two higher intensity feedback interventions with simple feedback on improving staff communication in nursing homes—the INFORM cluster-randomized controlled trial
Source: Implement Sci. 2020 Sep 10;15:75. doi: 10.1186/s13012-020-01038-3 (PMC7488270; doi:10.1186/s13012-020-01038-3)
Supplement: Supplementary file 1 — Additional file 1. Study measures. [file 13012_2020_1038_MOESM1_ESM.docx]

**Additional file 1: Study measures**

**Contents**

1. Primary study outcome: Formal Interactions 2
2. Secondary study outcomes 2
   1. Organizational context (modifiable features of the care unit work environment) 2
   2. Care unit characteristics 3
   3. Facility characteristics 3
   4. Staff outcomes 4
      1. Use of research-based best practices 4
      2. Health and quality of worklife 4
   5. Resident outcomes 5
      1. What are RAI quality indicators? 5
      2. What are practice sensitive RAI quality indicators? 5
      3. Two practice sensitive RAI quality indicators selected as secondary study outcomes 5
3. **Primary study outcome: Formal Interactions***(Part of the Alberta Context Tool, included in the Translating Research in Elder Care Care Aide Survey)*

| **Domain** | **Concept Definition** | **Scoring** | **Psychometric Properties** | **Sample Item** |
| --- | --- | --- | --- | --- |
| **Formal Interactions** | Formal exchanges that occur between individuals working within an organization (unit) through scheduled activities that can promote the transfer of knowledge. | 4 items scored on a 5-point Likert frequency scale: 1-never to 5-almost always.  Each item is recoded as: never/rarely = no interaction (0); occasionally = interaction (.5); yes = interaction (1). The overall score is a count taken of the 4 recoded items. | The Alberta Context Tool (ACT) consists of 10 concepts. The tool was originally developed in acute care, was adapted for long-term care and tested for reliability and validity with healthcare aides in TREC 1.0.^1^ Psychometric properties were also assessed for the nurse version of the ACT by combining data from multiple studies across settings (TREC 1.0 results were also included.^2^ These analyses supported that the ACT is an appropriate measure for assessing organizational context in nursing homes.  **Reliability**  In the care aide version, Cronbach's alpha for 8 of the 10 ACT concepts exceeded the commonly accepted standard of 0.70,^1^ and in the nurse version, Cronbach’s alpha for 9 of the 10 concepts exceeded 0.70.^2^  **Validity**  Confirmatory factor analyses of the care aide as well as the nurse version demonstrated that the overall pattern of the data tested was consistent with the structure hypothesized in the development of the ACT.^1,2^ Additionally, bivariate associations between the ACT concepts and instrumental research utilization (which the ACT was developed to predict) were statistically significant at the 5% level for 8 of the 10 ACT concepts in the care aide version. In addition, the majority (8/10) of the ACT care aide concepts showed a statistically significant trend of increasing mean scores when arrayed across the lowest to the highest levels of instrumental research use, further supporting its validity with healthcare aide ^1^. The mean values for each ACT concept in the nurse version increased from low to high levels of research utilization (as hypothesized) further supporting its validity.^2^ | *“In the past typical month, how often have you participated in a change of shift report?”* |

1. **Secondary study outcomes**
   1. **Organizational context (modifiable features of the care unit work environment)***(Part of the Alberta Context Tool, included in the Translating Research in Elder Care Care Aide Survey)*

| **Concept** | **Concept Definition** | **Scoring** | **Psychometric Properties** | **Sample Item** |
| --- | --- | --- | --- | --- |
| **Evaluation** | The process of using data to assess group/team performance and to achieve outcomes in organizations or units (i.e., evaluation). | 6 items scored on a 5-point Likert agreement scale: 1 = strongly disagree to 5 = strongly agree.  Overall score is derived by taking the mean of the scale items. | Concept of ACT, as above. | *“Our team routinely monitors our performance with respect to the action plans.”* |
| **Social Capital** | The stock of active connections among people. These connections are of three types: bonding, bridging, and linking. | 6 items scored on a 5-point Likert agreement scale: 1 = strongly disagree to 5 = strongly agree.  Overall score is derived by taking the mean of the scale items. | Concept of ACT, as above. | *“People in the group share information with others in the group.”* |
| **Organizational Slack: Time** | The cushion of actual or potential time resources which allows an organization (unit) to adapt successfully to internal pressures for adjustments or to external pressures for changes. | 4 items scored on a 5-point Likert frequency scale: 1 = never to 5 = almost always.  Overall score is derived by taking the mean of the scale items. | Concept of ACT, as above. | *“How often do you have time to do something extra for residents?”* |

- 1. **Care unit characteristics***(Translating Research in Elder Care Unit Survey)*

| **Concept** | **Concept Definition** | **Scoring** | **Psychometric Properties** | **Sample Item** |
| --- | --- | --- | --- | --- |
| **Responding to Major Near Misses** | A major near miss is an event that would have resulted in death or serious physical or psychological injury but did not because it was caught or because of good luck. | 12 questions related to how the unit responds to major near misses. Each question is treated as an individual item. No composite score is generated.  Question one is rated on a 5-point Likert-agreement scale: 1-strongly disagree to 5-strongly agree, including a 6-don’t know option  The other questions are rated on a four point Likert-frequency scale: 1-always or almost always to 4-never or almost never, including a 5-don’t know option | NA | *“Individuals involved in* ***MAJOR NEAR MISSES*** *on my unit have a quick and easy way to capture/report on what happened.”* |
| **Organizational Citizenship Behaviour**  (included in the TREC care aide survey) | The Organizational Citizenship Behaviour–Innovative Climate (OCB-O) scale has 4-items and reflects constructive efforts by care managers to identify and implement changes with respect to work methods, policies, and procedures to improve the situation and performance.^3^ | 4 item subscale scored on a 5-point Likert agreement scale: 1 = strongly disagree to 5 = strongly agree  A score for each subscale is derived by taking the mean of the 4 items. | **Reliability**  Cronbach’s alpha: 0.60 – 0.88^3^  **Validity**  Confirmatory factor analyses showed that the hypothesized three factor model provided a significantly better fit to the data than alternative models ^3^.  **Reliability** in our own study Cronbach’s alpha was 0.70 ^4^. This study also demonstrated **validity** of the OCB (factorial, concurrent, predictive validity). | *“I often suggest to my co-workers new ways about how to improve work on the unit.”* |

## Facility characteristics *(Translating Research in Elder Care Facility Survey)*

| **Concept** | **Concept Definition** | **Scoring** | **Psychometric Properties** | **Sample Item** |
| --- | --- | --- | --- | --- |
| **Performance Reports** | Receipt and perceived quality of various forms of performance data at the facility level. | Incidence reports (yes/no, and if yes: is quality of data appropriate? – yes/no).  Resident/family satisfaction reports (yes/no, and if yes: is quality of data appropriate? – yes/no).  RAI-MDS 2.0 (yes/no, and if yes: various 9 additional questions specifying use and appropriateness of the reports). | These items were developed and tested in TREC 1.0. Revisions to the response scale were made to add respondent’s assessment of the quality of the data they receive. | *“Are the facility level RAI-MDS 2.0 reports adequate for your needs?”* |
| **Quality Improvement Activities** | Reflects a facility’s involvement in quality improvement related activities and networks and provision of QI education | First question (number of quality improvement activities) is rated on a 4-point frequency scale: 1-no activities, 4-7+ activities.  The other 6 items are scored on a 4-point Likert frequency scale: 1 = never to 5 = almost always.  Overall score is derived by taking the mean of the scale items | These items are newly developed for use in TREC 2.0. | *“Meetings external to your nursing home on quality improvement (e.g., external QI teams)”* |

- 1. **Staff outcomes***(Translating Research in Elder Care Unit Survey)*

### **Use of research-based best practices**

| **Concept** | **Concept Definition** | **Scoring** | **Psychometric Properties** | **Sample Item** |
| --- | --- | --- | --- | --- |
| **Conceptual Research Utilization (CRU)**  (included in the TREC care aide survey) | The cognitive, reflective use of research (best practices) where the best practice knowledge may change one's opinion or mind set about a specific practice area but not necessarily one's direct actions. It is an indirect application of research findings. | 5 items scored on a 5-point Likert agreement scale: 1 = never to 5 = almost always.  Overall score is derived by taking the mean of the scale items. | The scale was developed and tested for reliability and validity with healthcare aides TREC 1.0.  **Reliability:** Cronbach's alpha exceeded the commonly accepted standard of 0.70.^5^  **Validity:** Context validity established with a formal content validity assessment using a panel of international experts. Cognitive debriefing interviews were used to refine the scale and determine fit between the items and healthcare aide responses. CFA supported a 1-factor solution. Other criterion-related validity analysis support the scale–see:^5^ Item Response Theory analysis further support validity and precision of the CRU scale.^6^ | *“How often did best practice knowledge about things like pain management and managing difficult behaviors raise your awareness about new ways to care for residents?”* |
| **Instrumental Research Utilization (IRU)**  (included in the TREC care aide survey) | The use of observable research-based practices when caring for residents. Practice may be guided by guidelines, protocols, routines, care plans or procedures that are based on research. | A single item scored on a 5-point Likert agreement scale: 1 = never to 5 = almost always. | The item, originally developed in acute care, was adapted for long-term care in TREC 1.0.  **Reliability:** Not applicable as is a single item  **Validity:** In TREC 1.0, bivariate associations between the ACT concepts and instrumental research utilization (which the ACT was developed to predict) were statistically significant at the 5% level for 8 of the 10 ACT concepts, supporting validity of both the ACT and the Instrumental Research Utilizations.^1^  In a recent systematic review of the psychometric properties of self-report research utilization instruments, Squires et al. reported that these specific single item measures of instrumental, persuasive, and overall research utilization were used in 10 published articles across a variety of healthcare settings. Validity evidence from all applicable sources (content, response processes, relationships with other variables) was reported in one or more of the published studies.^7^ | *“How often did you use this type of best practice knowledge [examples are provided in the stem] to provide resident care?”* |

### **Health and quality of worklife**

| **Concept** | **Concept Definition** | **Scoring** | **Psychometric Properties** | **Sample Item** |
| --- | --- | --- | --- | --- |
| **Psychological Empowerment**  ***Meaning***  (included in the TREC care aide survey) | The Psychological Empowerment scale is composed of 4 subscales: Meaning, Competence, Self-determination, and Impact. We use the Meaning subscale as secondary study outcome.  Psychological empowerment reflects an active orientation in which an individual wishes and feels able to shape his or her work role and context ^8^. It is a state rather than a trait and is specific to the work domain (e.g., is not generalizable to one’s life situations and roles). | 3 items scored on a 5-point Likert agreement scale: 1 = strongly disagree to 5 = strongly agree.  Overall score is derived by taking the mean of the scale items. | **Reliability** and **Validity** were supported in the index paper using a sample of workers outside of healthcare.^8^  This scale was adapted for healthcare aides in nursing homes and pilot tested in an Ontario sample in 2014 and field tested in Year 1 of TREC 2.0 using methods previously developed and successfully applied in TREC 1.0.^5,9^  **Reliability** of the 3 scales ranged from 0.80 – 0.92.^4^  The Ontario work with TREC cross-**validation** sample supported the hypothesized 4-factor model of Psychological Empowerment.^4^ | *“The work I do is important to me.”* |
| **Job Satisfaction**  (included in the TREC care aide survey) | We will use a positively worded version of The Michigan Organizational Assessment Questionnaire Job Satisfaction Subscale (MOAQ-JSS-3^10^)  This is a global job satisfaction measure that reflects affective components (i.e. one’s feelings about his/her job). | 3-item scored on a 5-point Likert-agreement scale: 1 = strongly disagree to 5 = strongly agree.  The overall score is derived by taking the mean of the scale items. | The MOAQ-JSS-3 is a reliable and construct-valid measure of global job satisfaction.^11^  This scale was adapted for healthcare aides in nursing homes and pilot tested in an Ontario sample in 2014 and field tested in Year 1 of TREC 2.0 using methods previously developed and successfully applied in TREC 1.0.^5,9^  The Ontario validation work supported **validity** and **reliability** (Cronbach’s alpha = 0.8) of this modified job satisfaction scale.^4^ | *“All in all, I am satisfied with my job.”* |

## Resident outcomes *(Resident Assessment Instrument – Minimum Data Set 2.0; RAI-MDS 2.0)*

- - 1. **What are RAI quality indicators?**

A quality indicator (QI) is a computed measure (an event rate) based on a clinical outcome that is believed to be reflective of the quality of care. Outcomes can be undesirable, such as falls or pressure ulcers, or they may be desirable such as physical independence or improved continence. QI’s were central in the original conceptualization of the RAI-MDS assessment system, and public reporting of QI’s has been done for many years in the US, and it is beginning to be used in Ontario long-term care. Public reporting is thought to be a driver of improved quality either through consumer empowerment, or by ‘naming and shaming’. But more importantly, quality indicators give individual facilities or operators a standardized and comparable measure by which to target and monitor quality improvement activities. When reported with transparency, poor performers can identify facilities with good performance, and seek to learn from them. Researchers can use QIs as a metric to shed light on the effects of ownership, funding, policy, or care culture.

Some quality indicators are strictly cross-sectional (*e.g.,* use of indwelling catheters), while others use two consecutive assessments to identify individual-level improvement or decline. Central to QI construction is the issue of risk adjustment, which arises from understood risk factors associated with poor outcomes, and these risk factors being unevenly distributed among facilities. Methods for developing RAI-MDS-based QIs for use in nursing homes have been developed by the Centers for Medicare and Medicaid (CMS) in the US^12^ and have been applied in Canadian complex continuing care settings and long term care;^13^ more recently, the Canadian Institute for Health Information (CIHI) has developed third generation QIs.^14,15^

- - 1. **What are practice sensitive RAI quality indicators?**

There are 35 QI’s in the RAI system that use 3^rd^ generation risk adjustment,^14^ however not all of them are equally sensitive to changes in practice, be it nursing, medical, allied or combined interventions. As our intent in the TREC program is to work with *modifiable* outcomes, we have developed a set of what we term *practice sensitive QI’s*.^16^ The idea of working with modifiable QI’s is relevant to all study parts of TREC – given its mission to provide practical, sustainable solutions to improve quality of care and quality of life of frail, vulnerable nursing home residents. We developed a list of practice sensitive QI’s by first considering the list of 35 CIHI-supported indicators.^16^ Based on the opinions of two experts (Poss^17,18^ and Hirdes^19-22^) familiar with the selection and construction of these indicators, a list of 10 nursing practice, 2 physician practice, and 1 policy/legislation sensitive indicators were chosen. We then submitted this list to three groups of people – practising physicians, practising nurses and practising decision/policy-makers, all in the field of residential long LTC and geriatrics, and asked them to rank the list for both its overall “practice sensitivity” and then the domain to which it was most sensitive (nursing, medicine, policy).

### **Two practice sensitive RAI quality indicators selected as secondary study outcomes**

Note: Here CMS is the US Centre for Medicare and Medicaid Services. HQO is Health Quality Ontario. Both are listed as major agencies that have chosen these quality indicators for public reporting purposes, and as such have been judged to be both important indicators and with sufficient validity.

see:

<http://www.medicare.gov/NursingHomeCompare>

<http://www.hqontario.ca/en/reporting/ltc/>

| **Practice sensitive RAI QI** | **Evidence of QI Validity** |
| --- | --- |
| Worsening pain | - HQO: pain that recently got worse *(the new CMS QI for pain is based on the self-report item of the newer MDS 3.0, and not the MDS 2.0)* - The RAI-MDS *pain* QI has been found to accurately differentiate the prevalence of pain between facilities however it has been suggested that high pain prevalence scores were associated with more frequent pain assessment and appropriate pain-related care practices, as opposed to poor care quality.^23^ |
| Declining behavioral symptoms | There is little yet reported to support the validity of this indicator, however it is clinically important, associated with resident safety, and actionable by direct care providers. |

**References**

1. Estabrooks C, Squires J, Hayduk L, Cummings G, Norton P. Advancing the argument for validity of the Alberta Context Tool with healthcare aides in residential long-term care. *BMC Medical Research Methodology* 2011; **11**: 107.

2. Squires JE, Hayduk L, Hutchinson AM, et al. Reliability and validity of the Alberta Context Tool (ACT) with professional Nurses: Findings from a multi-study analysis. *PLoS One* 2015; **10**(6): e0127405.

3. Choi JN. Change-oriented organizational citizenship behavior: effects of work environment characteristics and intervening psychological processes. *Journal of Organizational Behavior* 2007; **28**(4): 467-84.

4. Ginsburg L, Berta W, Baumbusch J, et al. Measuring Work Engagement, Psychological Empowerment, and Organizational Citizenship Behavior Among Health Care Aides. *Gerontologist* 2016; **e-pub. ahead of print**.

5. Squires J, Estabrooks CA, Newburn-Cook C, Gierl M. Validation of the Conceptual Research Utilization Scale: An application of the Standards for Educational and Psychological Testing in Healthcare. *BMC Health Services Research* 2011; **11**: 107.

6. Squires J, Estabrooks CA, Hayduk L, Gierl M, Newburn-Cook CV. Precision of the Conceptual Research Utilization Scale. *J Nurs Meas* 2014; **22**(1): 145-63.

7. Squires J, Estabrooks C, O'Rourke H, Gustavsson P, Newburn-Cook C, Wallin L. A systematic review of the psychometric properties of self-report research utilization measures used in healthcare. *Implementation Science* 2011; **6**: 83.

8. Spreitzer GM. Psychological Empowerment in the Workplace: Dimensions, Measurement, and Validation. *The Academy of Management Journal* 1995; **38**(5): 1442-65.

9. Squires JE, Kong LL, Brooker S, Mitchell A, Sales AE, Estabrooks CA. Examining the Role of Context in Alzheimer Care Centers: A Pilot Study. Edmonton, AB: Knowledge Utilizations Studies Program, Faculty of Nursing, University of Alberta, 2009.

10. Bowling NA, Hammond GD. A meta-analytic examination of the construct validity of the Michigan Organizational Assessment Questionnaire Job Satisfaction Subscale. *J Vocat Behav* 2008; **73**(1): 63-77.

11. Schleicher DJ, Hansen D, Fox KE. Job attitudes and work values. In: Zedeck S, ed. APA handbook of industrial and organizational psychology, Vol 3. Washington, DC: APA; 2010: 137-90.

12. Berg K, Mor V, Morris J, Murphy KM, Moore T, Harris Y. Identification and evaluation of existing nursing homes quality indicators. *Health Care Financing Review* 2002; **23**(4): 19-36.

13. Wodchis WP, Teare GF, Anderson GM. Cost and Quality: Evidence From Ontario Long Term Care Hospitals. *Medical Care* 2007; **45**(10): 981-8 10.1097/MLR.0b013e3180ca95e9.

14. Canadian Institute for Health Information. Continuing care reporting system RAI-MDS 2.0 output specifications, 2014–2015. Ottawa, ON: CIHI; 2015.

15. Jones RN, Hirdes JP, Poss JW, et al. Adjustment of nursing home quality indicators. *BMC Health Serv Res* 2010; **10**(1): 96.

16. Estabrooks CA, Knopp-Sihota JA, Norton PG. Practice sensitive quality indicators in RAI-MDS 2.0 nursing home data. *BMC Res Notes* 2013; **6**(1): 460.

17. Poss J, Murphy K, Woodbury M, et al. Development of the interRAI Pressure Ulcer Risk Scale (PURS) for use in long-term care and home care settings. *BMC Geriatrics* 2010; **10**: 67.

18. Poss JW, Hirdes JP, Fries BE, McKillop I, Chase M. Validation of Resource Utilization Groups Version III for Home Care (RUG-III/HC): Evidence From a Canadian Home Care Jurisdiction. *Medical Care* 2008; **46**(4): 380-7.

19. Hirdes J, Ljunggren G, Morris J, et al. Reliability of the interRAI suite of assessment instruments: a 12-country study of an integrated health information system. *BMC health services research* 2008; **8**(277).

20. Hirdes J, Poss J, Curtin-Telegdi N. The Method for Assigning Priority Levels (MAPLe): A new decision-support system for allocating home care resources. *BMC Medicine* 2008; **6**: 9.

21. Hirdes JP, Fries BE, Morris JN, et al. Home Care Quality Indicators (HCQIs) Based on the MDS-HC. *The Gerontologist* 2004; **44**(5): 665-79.

22. Hirdes JP, Frijters DH, Teare GF. The MDS-CHESS scale: A new measure to predict mortality in institutionalized older people. *Journal of the American Geriatrics Society* 2003; **51**(1): 96-100.

23. Cadogan MP, Schnelle JF, Yamamoto-Mitani N, Cabrera G, Simmons SF. A Minimum Data Set Prevalence of Pain Quality Indicator: Is It Accurate and Does It Reflect Differences in Care Processes? *The Journals of Gerontology Series A: Biological Sciences and Medical Sciences* 2004; **59**(3): M281-M5.
